# “Men are always scared to test with their partners … it is like taking them to the Police”: Motivations for and barriers to couples’ HIV counselling and testing in Rakai, Uganda: a qualitative study

**DOI:** 10.7448/IAS.17.1.19160

**Published:** 2014-09-18

**Authors:** Joseph KB Matovu, Rhoda K Wanyenze, Fred Wabwire-Mangen, Rosette Nakubulwa, Richard Sekamwa, Annet Masika, Jim Todd, David Serwadda

**Affiliations:** 1 Department of Community Health & Behavioral Sciences, School of Public Health, Makerere University, Kampala, Uganda; 2 Department of Disease Control & Environmental Health, School of Public Health, Makerere University, Kampala, Uganda; 3 Regional Center for Quality of Health Care, School of Public Health, Kampala, Makerere University, Uganda; 4 Department of Qualitative Research, Rakai Health Sciences Program, Kalisizo, Uganda; 5 Department of Population Health, London School of Hygiene and Tropical Medicine, London, United Kingdom

**Keywords:** Motivations, barriers couples, counselling, testing, Rakai, Uganda

## Abstract

**Introduction:**

Uptake of couples’ HIV counselling and testing (couples’ HCT) can positively influence sexual risk behaviours and improve linkage to HIV care among HIV-positive couples. However, less than 30% of married couples have ever tested for HIV together with their partners. We explored the motivations for and barriers to couples’ HCT among married couples in Rakai, Uganda.

**Methods:**

This was a qualitative study conducted among married individuals and selected key informants between August and October 2013. Married individuals were categorized by prior HCT status as: 1) both partners never tested; 2) only one or both partners ever tested separately; and 3) both partners ever tested together. Data were collected on the motivations for and barriers to couples’ HCT, decision-making processes from tested couples and suggestions for improving couples’ HCT uptake. Eighteen focus group discussions with married individuals, nine key informant interviews with selected key informants and six in-depth interviews with married individuals that had ever tested together were conducted. All interviews were audio-recorded, translated and transcribed verbatim and analyzed using Nvivo (version 9), following a thematic framework approach.

**Results:**

Motivations for couples’ HCT included the need to know each other's HIV status, to get a treatment companion or seek HIV treatment together – if one or both partners were HIV-positive – and to reduce mistrust between partners. Barriers to couples’ HCT included fears of the negative consequences associated with couples’ HCT (e.g. fear of marital dissolution), mistrust between partners and conflicting work schedules. Couples’ HCT was negotiated through a process that started off with one of the partners testing alone initially and then convincing the other partner to test together. Suggestions for improving couples’ HCT uptake included the need for couple- and male-partner-specific sensitization, and the use of testimonies from tested couples.

**Conclusions:**

Couples’ HCT is largely driven by individual and relationship-based factors while fear of the negative consequences associated with couples’ HCT appears to be the main barrier to couples’ HCT uptake in this setting. Interventions to increase the uptake of couples’ HCT should build on the motivations for couples’ HCT while dealing with the negative consequences associated with couples’ HCT.

## Introduction

There is growing evidence that HIV infections are declining in most parts of the world, including sub-Saharan Africa, with declines of 50% or more reported in 25 middle- and low-income countries between 2001 and 2011 (1). However, despite a 25% decline in HIV incidence in sub-Saharan Africa over the same period, the number of new HIV cases that occurred in this region during the same 10-year period accounted for 72% of all new HIV infections globally (1). Modelling studies suggest that infections occurring among married and cohabiting couples contribute between 40 and 60% of the total HIV incidence (2, 3), while Dunkle *et al*. (4) found that 55–93% of new HIV infections occurring in urban Rwanda and Zambia originated from sero-discordant marital or cohabiting couples. The high cases of new HIV infections occurring among couples poses a great public health challenge considering that up to 60% or even more of the adult population in sub-Saharan Africa is married or cohabiting with regular partners (5). As new HIV infections among couples rise, so do HIV transmission possibilities, including the possibility of intra-couple HIV transmission among HIV-discordant couples, extra-couple transmission when HIV-positive married individuals have sex with non-marital partners and parent-to-child transmission of HIV. These findings suggest that interventions targeting couples in stable partnerships, including couples’ HIV counselling and testing (couples’ HCT) (6), can have a substantial impact on population-level HIV incidence.

Modelling studies suggest that targeting HIV-discordant couples with appropriate interventions, including couples’ HCT, can avert between 36 and 60% of heterosexually transmitted infections that would otherwise occur in the absence of any interventions (4). In addition, recent clinical trials suggest that timely initiation of anti-retroviral therapy among HIV-discordant couples can reduce HIV transmission from the infected to the uninfected partner by up to 96% (7). There is also evidence to show that testing together can lead to reductions in sexual risk-taking behaviours among HIV-discordant couples 8–(10) , facilitate early identification of previously undiagnosed HIV infections among couples (11) and improve timely linkage to and retention in HIV care (12). Couples’ HCT can also benefit HIV-negative couples by reducing mistrust and improving prospects for joint plans on how to avoid HIV infection (12). However, while the benefits of couples’ HCT are evident, majority of couples have never tested together let alone know each other's HIV status 13–(15) .

Several reasons have been cited to explain the low uptake of couples’ HCT. These reasons include fear of marital consequences (such as intimate partner violence or marital dissolution) following couples’ HCT (16, 17); low male participation (18) largely driven by fears among men that couples’ HCT could expose hidden infidelity (19, 20); and the perception that monogamy is safe (despite high levels of new HIV infections occurring among couples) coupled with beliefs in HIV testing by proxy (18, 21). High levels of mistrust between partners (22) largely driven by concerns about partner infidelity (23); male dominance in decision-making (24) fuelled by gender and power dynamics that tend to favour men over women (20, 25); and health system factors such as lack of male-friendly services at antenatal clinics (22, 26) and the belief that HIV testing is a women's preserve (18, 20) have also been found to limit couples’ HCT uptake.

Although attempts have been made to promote couples’ HCT through antenatal care and prevention of mother-to-child transmission (PMTCT) of HIV programmes that encourage HIV testing of pregnant women with their male partners (13, 26) (27), the promotion of couples’ HCT tested at these sites has not improved over time. In a study by Byamugisha *et al*. (26) in eastern Uganda, only 5% of pregnant women were tested with their male partners during antenatal care, while another study by Becker *et al*. (14) in Tanzania indicated that only 16% of couples received couples’ HCT. Outside antenatal clinics, uptake of couples’ HCT shows a promising trend (15, 27), with uptake rates ranging between 47 and 62% in home-based HCT studies (15, 28) although lower uptake rates have been reported in some studies (29, 30). However, because the majority of couple studies have been conducted at antenatal or PMTCT sites (13, 14) (24), or among HIV-discordant couples (8, 31) rather than in the general population, our understanding of the motivations for and barriers to couples’ HCT in a general population context remains largely limited.

In this paper, we present findings from a qualitative study conducted to explore motivations for and barriers to couples’ HCT uptake among married individuals and selected key informants in a population-based cohort in Rakai, Uganda. This study was informed by theoretical constructs from the Health Belief Model (perceived benefits and perceived barriers) (32) and was intended to generate data necessary to inform the design and implementation of a demand creation intervention aimed at improving couples’ HCT uptake among married couples in Rakai, Uganda.

## Methods

### Study site

This study was conducted in three purposively selected study clusters that were selected from three geographical strata (i.e. rural communities [Katana]; main-road communities [Kasasa-Sanje]; and fishing villages [Kasensero]) based on HIV prevalence data (33). The selection of the three clusters was informed by the need to assess the impact of the intervention in areas of differing HIV prevalence so as to improve applicability of study findings across a diversity of settings. The three study clusters are part of the Rakai community cohort, a population-based cohort that was established in Rakai district in 1994 (34). The main study, the Rakai Community Cohort Study (RCCS), enrols consenting individuals aged 15–49 years, who are resident in these study clusters, through annual study visits. Free HCT services are available to study participants within the study communities; and individuals can elect to receive their HIV test results alone or together with their partners (35).

### Study design

This was a qualitative study that used focus group discussions (FGDs), in-depth interviews (IDIs) and key informant interviews (KIIs) to explore motivations for and barriers to couples’ HCT in Rakai district. FGDs were considered appropriate for generating data on community perceptions regarding the motivations for and barriers to HCT uptake among married individuals as well as for soliciting views on what needs to be done to improve couples’ HCT uptake in the community. IDIs were considered appropriate for generating data on personal, lived experiences of married individuals that had ever received couples’ HCT. KIIs were considered appropriate for generating data on general perceptions about couples’ HCT uptake and what needs to be done to improve uptake, from the point of view of “community gatekeepers.”

### Selection of study communities

Each of the three study clusters is composed of three to seven study communities. One study community each was purposively selected from each cluster to participate in the study, and within each study community, three villages were selected. In total, nine villages (three in each study cluster) were selected to participate in this study.

### Study participants

Study participants were married individuals and selected key informants (“community gatekeepers”) who were resident in selected villages within each study cluster. Married individuals were defined as men and women who were living together as husband and wife following a religious, civil, traditional or consensual marriage ceremony.

### Participant selection

#### FGD participants

FGD participants were married individuals who had participated in previous RCCS study visits at least once. Participation in the FGDs did not require that the individual participants come from the same marital union. Participants were selected from the RCCS database, with permission from the Rakai Health Sciences Program (the programme that conducts the RCCS). Initially, we requested the data manager to generate a list of all married individuals for whom complete information on partner, HIV and prior HCT status was available. Using HCT status information, we categorized married individuals into three distinct categories: 1) both partners had never tested for HIV; 2) one or both partners had ever tested for HIV separately (i.e. not together with their partner); or 3) both partners had ever tested and received their HIV test results together (i.e. received couples’ HCT). We then requested the data manager to print lists of all selected individuals and pass them on to community health mobilizers (CHMs) to notify participants at least three days prior to the scheduled interview. CHMs are community volunteers who work with the RCCS team to locate study participants in the community. To maximize confidentiality and improve prospects for participation, the lists of all respondents that were sent to CHMs did not contain HCT or HIV status information. Six FGDs were conducted for each HCT category per study cluster for a total of 18 FGDs in the three clusters. FGDs were conducted at community venues (such as schools, community halls or church compounds) that offered free interaction with the participants while increasing prospects for confidentiality of information shared with the study team. Each FGD was facilitated by two trained research assistants (a moderator and a note-taker) and all interviews were audio-recorded with consent from the participants. Overall, 142 individuals participated in FGDs in the three study clusters. Each FGD participant received 7000 Uganda shillings (approx. US$2.8, based on 2013 exchange rates) to compensate for their time as well as cover travel costs incurred.

#### IDI & KII participants

IDI participants were married individuals who had ever received couples’ HCT, selected from the RCCS database. Prior to their selection, we asked the data manager to form “couples” by linking all married individuals to their spouses using a study identifier. We then asked the data manager to link all “couples” with the HIV information that was already available in the RCCS database. This enabled him to assign an HIV status to each member of the couple. Couples were categorized as HIV-discordant if one of the partners was HIV-positive while the other was not; HIV-positive if both partners had HIV; and HIV-negative if both partners did not have HIV. Only individuals who had ever received couples’ HCT were selected to participate in IDIs. Overall, six IDIs were conducted in the three study clusters. On the contrary, KII participants were community residents who normally interact with married individuals and who could influence utilization of HCT services in their respective communities. These included CHMs, HIV counsellors, and religious leaders. Prior to their selection, the study team generated names of individuals that could be approached in each category and assigned one of the research assistants to contact them and seek their willingness to participate in the study. Those that were interested in participating in the study were informed about the date and time of the interview. Overall, nine key informants were interviewed in the three study clusters. Both IDIs and KIIs were conducted at venues (such as the participant's home) that were agreeable to the participant and the interviewer, as long as such venues guaranteed confidentiality and offered participants an opportunity for free discussion. Each IDI or KII was facilitated by one research assistant and audio-recorded with consent from the participant. IDI and KII participants provided written informed consent prior to participating in the study, and each participant received 5000 Uganda Shillings (approx. US$2, based on 2013 exchange rates) as compensation for time, at the end of the interview.

### Data collection procedures

Data collection took place between August and October 2013. Data were collected by a team comprising the first author and three research assistants working with the Qualitative Research Unit of the Rakai Health Sciences Program. All research assistants, who are degree holders with substantive experience in the design and conduct of qualitative research, received orientation on the objectives of the study and were trained in how to administer the study tools. They then participated in the piloting of interview tools (in non-study communities) to gain further insights into the interpretation and flow of questions, as well as to master the primary purpose of the study. Data were collected on motivations for couples’ HCT, barriers to couples’ HCT; decision-making processes and experiences from tested couples; and suggestions for improving couples’ HCT uptake in Rakai. Prior to each interview, we collected socio-demographic data (age, sex, education, marital duration) from each participant to help us characterize the individuals participating in the study.

Data collection was done using pilot-tested, unstructured interview guides. Interviews took between 1 and 2 hours. Similar questions were administered to all participants across the different study clusters and population sub-groups except that an additional module of questions was administered to IDI participants in order to capture their experiences pertaining to couples’ HCT uptake. Interviews were conducted in the local language (*Luganda*), audio-taped, translated and transcribed verbatim by trained research assistants within 12 hours of data collection. In addition, detailed hand-written notes were taken to supplement data captured on the audio-recorder. After each data collection exercise, debriefing meetings were held with the research assistants to ensure good-quality data and share new and emerging issues.

### Data analysis

Data analysis was performed using Nvivo (version 9), following a thematic framework approach (36). Initially, the research assistants independently read through transcribed texts using a data analysis template developed by the first author to identify issues pertaining to each theme. This resulted in data analysis tables that were arranged by study cluster and thematic area. This helped us to track the extent to which issues emerging from each theme were common across clusters and thematic areas. The analysis tables were then reviewed by the first author to ensure completeness of data retrieval as well as check on the labelling of issues identified for each theme. The research assistants again read through all the issues identified for each theme, noting any emerging issues while generating codes along the way. This process was continued until no new issues emerged/codes were generated (see Table 2 for a list of issues/codes that emerged during the analysis). Using a coding scheme, we coded and managed the data using Nvivo version 9 and exported all coded sections into MS Word for additional analysis. Finally, we read through the text to identify relevant quotes, based on the issues/codes generated, to illustrate participants’ views. It is important to note that where names have been mentioned in the quotation, these are not the real names of participants but pseudonyms created for reporting purposes only. The names of places shown against each quotation pertain to the names of entire study clusters rather than specific names of villages where interviews were conducted.

### Ethical considerations

The study was approved by the Higher Degrees, Research and Ethics Committee of Makerere University School of Public Health and the Uganda National Council for Science and Technology.

## Results

### Participants’ characteristics

One hundred and forty two married individuals (50% male) participated in 18 FGDs conducted in three study clusters in the Rakai district (Table 1). The majority of the participants were aged between 25 and 44 (82%); 60% had primary education, while 60% had been married for 10 or more years. Of the 142 participants, 32% had never tested for HIV; 33% had ever tested and received their HIV test results separately; while 35% had ever tested and received their HIV test results together with their partners. Key informants and IDI participants were aged between 31 and 60, primarily men (11 of 15 participants), with primary or higher education, and married for 10 or more years. Overall, 157 individuals participated in the FGDs, KIIs and IDIs.

**Table 1:** Study participants, data collection methods and study clusters

Characteristic	Kasensero	Buyamba	Kasasa-Sanje	Total

Focus group discussions (FGDs)				
No. of FGDs	06	06	06	18
Total interviewed	44	42	56	142
Gender (%)				
Male	22 (50.0)	21 (50.0)	28 (50.0)	71 (50.0)
Female	22 (50.0)	21 (50.0)	28 (50.0)	71 (50.0)
Age group (%)				
15–24	06 (13.6)	05 (11.9)	01 (1.8)	12 (8.5)
25–34	19 (43.2)	14 (33.3)	23 (41.1)	56 (39.4)
35–44	17 (38.6)	17 (40.5)	27 (48.2)	61 (43.0)
45+	02 (4.5)	06 (14.3)	05 (8.9)	13 (9.1)
Education (%)				
No education	12 (27.3)	04 (9.5)	05 (8.9)	21 (14.8)
Primary	24 (54.5)	27 (64.3)	35 (62.5)	86 (60.6)
Secondary	08 (18.2)	11 (26.2)	13 (23.2)	32 (22.5)
Tertiary	–	–	03 (5.4)	03 (2.1)
Marital duration (%)				
<1 year	01 (2.3)	–	–	01 (0.7)
1–4 years	16 (36.4)	03 (7.1)	03 (5.4)	22 (15.5)
5–9 years	13 (29.5)	10 (23.8)	11 (19.6)	34 (23.9)
10+ years	14 (31.8)	29 (69.0)	42 (75.0)	85 (59.9)
HCT status (%)				
Never tested	14 (31.8)	15 (35.7)	16 (28.6)	45 (31.7)
Received individual HCT	15 (34.1)	14 (33.3)	18 (32.1)	47 (33.1)
Received couples’ HCT	15 (34.1)	13 (31.0)	22 (39.3)	50 (35.2)
Key informant interviews (KIIs)				
No. of KIIs	03	03	03	09
HIV counsellor	01	01	01	03
Community health mobilizer (CHM)	01	01	01	03
Religious leader	01	01	01	03
In-depth interviews (IDIs)				
No. of IDIs	02	02	02	06
Individuals that had ever received couples’ HCT	02	02	02	06

### Motivations, couples’ HCT experiences, barriers and suggestions for improving couples’ HCT uptake

The findings have been grouped into four a priori themes as follows: 1) motivations for couples’ HCT; 2) decision-making process and experiences from tested couples; 3) barriers to couples’ HCT, and 4) suggestions on how couples’ HCT uptake can be improved. The findings pertaining to each of these themes are summarized in Table 2 below as well as in the sub-sections that follow.

**Table 2:** Themes and key issues on the motivations for and barriers to couples’ HIV counselling and testing in Rakai, Uganda

Theme	Issue

1.0 Motivations for couples’ HIV counselling and testing	1.1 Know each other's HIV status 1.2 Linkage to HIV care and identification of treatment reminders 1.3 Reducing mistrust and improving marital relationships 1.4 Planning for the future of family and/or children 1.5 Couples tested together can live happier and healthier lives
2.0 Barriers to couples’ HIV counselling and testing	2.1 Fear of receiving concordant HIV-positive or HIV-discordant results 2.2 Fear of marital violence or dissolution 2.3 Fear that couples’ HCT could expose hidden infidelity 2.4 Fear of being ashamed before one's partner in the event of an HIV-positive status 2.5 Urge by one or both partners to hide HIV status from each other 2.6 Lack of trust/misunderstandings in the home 2.7 Men's reluctance/refusal to test for HIV together with their partners 2.8 Conflicting schedules between men and women
3.0 Decision-making process and experiences from tested couples	3.1 Partner tested alone initially before inviting the other partner to test together 3.2 Initial resistance from invited partner 3.3 HIV-negative partners in HIV-discordant relationships were initially disturbed by the sero-positive status of their partners 3.4 Enriching and fulfilling experiences for concordant HIV-negative partners
4.0 Suggestions for improving couples’ HIV testing	4.1 Hold couple-specific meetings to sensitize couples on the benefits of couples’ HIV testing 4.2 Send invitation letters to couples 4.3 Promote couples’ HCT by going to people's homes 4.4 Use religious leaders to sensitize their flock about couples’ HCT 4.5 Provide preferential treatment to couples when they come to test for HIV together 4.6 Use expert couples, i.e., couples that have ever tested together to motivate others to test for HIV together

#### Motivations for couples’ HCT

Across all interviews – FGDs, KIIs and IDIs – the most commonly cited motivation for couples’ HCT was the need to know each other's HIV status (Table 2, issue# 1.1). Knowing each other's HIV status was viewed as important for couples to initiate HIV treatment if one or both partners were HIV-positive, and for enhancing behaviour change based on the results received:

What motivated us was to know the truth and to see whether we are not infected with HIV or … if we are HIV positive we can start on HIV drugs when it's still early. If we are HIV negative we should continue protecting ourselves so that we don't acquire the virus (FGD, women, ever received HCT as a couple, Buyamba)

Participants indicated that in the event that one of the partners were HIV-positive, receiving HCT together would provide them with greater opportunity to remind the HIV-positive partner about taking their HIV treatment on time, or collect drugs from the health facility on their behalf in the event that they are unable to do so (Table 2, issue# 1.2). This would be equally true if both partners were HIV-positive: if they knew each other's HIV status, this would help them to remind each other about taking their treatment on time, or collect each other's HIV drugs from the health facility.

There was a general agreement among all participants that couples’ HCT can help to reduce mistrust between partners (Table 2, issue# 1.3) and also could serve as a way of building trust and increasing faithfulness between partners, particularly among those that are concordant HIV-negative. To men, couples’ HCT was viewed as an opportunity to be absolved from the accusations of infidelity by their female partners:

Our wives don't believe/trust in us and are always suspicious of us. They do say that we have other sexual partners outside there. So, if we go together for HIV counseling and testing she begins to trust in me and will no longer be suspicious of me having other sexual partners and this can help her to have a settled mind (FGD, men, never received HCT, Buyamba).

Participants viewed trust in terms of sexual fidelity: in most cases, whenever they referred to lack of trust, it was because one or both partners were engaged in extra-marital relations. Across all the interviews, there was a general perception that it is the trust that partners have for each other that can eventually build into the motivation to seek couples’ HCT:

One thing which motivates couples is the trust they have for one another … People behave differently. We marry women who are totally different. You do not know where she comes from and you do not know her parents. Gradually, you trust this woman and turn her into a wife. Once there is trust then you ask to go and test with her (IDI with HIV positive man who received couples’ HCT in a concordant HIV-positive relationship, Kasensero)

The need to plan for the future of their families and children emerged as another important motivator for couples to test for HIV together (Table 2, issue# 1.4). Participants referred to the fact that couples’ HCT allows partners to know their status and if one of them were HIV-positive, it would help the HIV-negative partner to remain HIV-free in order to *be able to provide parental support to our children when my spouse passes away*. Although concerns about the future of children were more commonly expressed by female FGD participants, similar views were also raised by male participants and key informants in the different interviews held across the three study clusters. This suggests that planning for the future of families, and particularly the future of children, is an important motivator for couples’ HCT.

There was also a perception that individuals who tested together tended to live happier and healthier lives than those who had never tested together, especially if one or both of them were enrolled in HIV care (Table 2, issue# 1.5). Although this perception was not widespread (it was only captured in Kasasa-Sanje), it is likely that the belief that couples enrolled in HIV care tended to live happier and healthier lives could be a motivator for untested couples to seek joint HCT services, as the following quotation illustrates:

I have seen couples who tested and were both HIV positive. I see how they behave and what developments they do. I have seen them live longer than those who failed to test yet they had signs of HIV. I have seen that couples who tested together are happy and always do several things together but those that failed to test are always quarreling and fighting (FGD, men, ever received couples’ HCT, Kasasa-Sanje)

#### Barriers to couples’ HCT

When asked about the main barriers to couples’ HCT uptake, the majority of the participants cited fear of the negative consequences arising from receiving couples’ HCT to be the single most important barrier to testing as a couple (Table, issue# 2.1–2.4). Fear was manifested in several forms including fear of receiving HIV-discordant or concordant HIV-positive results, fear of marital violence or marital dissolution, fear of being blamed as being responsible for bringing HIV into the family and fear that couples’ HCT could expose a partner's hidden engagement in extra-marital relations:

The first thing that stops couples from going for HIV testing together with their partners is the fear to get HIV positive results. Most people say that if I go to test for HIV with my partner they can tell me that I have HIV and this can make me have a lot of worries and stress. So to avoid this stress and worries, someone decides not to go for HIV counseling and testing [together with their partners] (KII with a religious leader, Kasensero)

Behind the stress and worries were fears of marital dissolution that people constantly referred to, especially in the event that one of the partners, and especially the female partner, was HIV-positive. Since marital relationships are formed around men who assume the head of the household role, women are often in constant fear of being chased out of the home if they are found to be HIV-positive:

Some women fear that if their husbands find out that they have HIV, they can tell them to go away from their homes. You know women move from their parents’ homes to come and stay with their husbands, so a man might decide to chase you away from his home if he finds that she has HIV. Therefore, they fear to be chased away from their marriage after testing HIV positive and this makes it difficult for them to go for HIV testing together with their partners. (KII with a religious leader, Kasensero)

Beyond the fears of the negative consequences associated with couples’ HCT, we found that some participants did not want their partners to know their HIV status and this, in a way, acted as a barrier to joint HCT. We found that the urge to hide one's HIV status was common among men who, possibly because of sexual infidelity, would rather keep their HIV status, especially if HIV-positive, secret rather than disclose it through couples’ HCT (Table 2, issue# 2.5). While the issue of hiding HIV status was not commonly expressed in other study clusters other than Kasensero, we thought it was important to highlight it as a key deterrent to couples’ HCT uptake, given that it is connected to the other barriers such as fears of being blamed for having brought the infection into the family or fears of being chased out of the home, in the case of women. We noted that the urge to hide HIV status from the partner was more common in couples where there were suspicions of sexuality infidelity.

Suspicions of sexual infidelity usually result in high levels of mistrust or misunderstandings between partners which in themselves have been reported as deterrents to the uptake of couples’ HCT (Table 2, issue# 2.6). Across interviews, there was a general perception that when there is no love between the partners (due to mistrust), it is hard for them to go together to test for HIV:

[…] if there is no love amongst the couples, there is no way one of them will say that we go for an HIV test. In some homes, you find that the couple is not getting on well and you find that they have their internal wrangles as a couple and don't get on well at home. So this will make it hard for one of them to convince the other that we go for an HIV test together (KII, religious leader, Kasensero)

In Rakai, as in other settings, men fear to go for HIV testing together with their partners because they think this could not only reveal cases of hidden infidelity (Table 2, issue# 2.7) but also make them ashamed of their HIV status if they are found to be HIV-positive when their partners are HIV-negative:

[…] you can find that a man is having like ten other sexual partners yet he might not know their HIV status. So this makes him fear to go for an HIV test with his wife because she might find out that her husband has HIV and become ashamed in front of her and because of this fear to get ashamed in front of his wife, he can decide not to go for an HIV test with her but rather go there when he is alone. (KII with a religious leader, Kasensero)

The fear of being ashamed before one's partner manifested itself in the form of men's pretense that they were “busy” and did not have time to go for HIV testing together with their female partners. In such cases, men tended to ask their female partners to go for HIV testing rather than go together with them in the hope that the female partners’ HIV status could be assumed to be the same as theirs. In one particular scenario, a key informant equated men's fears of taking an HIV test together with their female partners as similar to *being taken to the Police*:

Again men have fears because their partners are always suspicious of their sexual behaviors (men's sexual behaviors). You hear women blaming their partners about being promiscuous. Men are always scared to test with their partners because it is like taking them to the Police [providing evidence for their promiscuous sexual behaviors] (KII with HIV counsellor, Buyamba)

Long distances to the testing facility, the cost of testing both partners, couples’ lack of knowledge about where to go for couples’ HCT and conflicting work schedules between partners were highlighted as other critical barriers to the uptake of couples’ HCT. The issue of conflicting work schedules was particularly brought out by participants at landing sites who indicated that men tend to “spend days in the lake” and are therefore not at home for most of the time to receive HCT together with their female partners (Table 2, issue# 2.8). However, this issue also applied to other people working in other settings, as the quotation below illustrates:

[…] there are some men who work in different places and are always at their place of work most of the time and they go to see their partners only on weekends yet they might be having a busy schedule on the weekend thus not finding time to go for an HIV test together with their wives (KII with a religious leader, Kasensero)

#### Decision-making process and experiences from tested couples

Experiences from those that had ever received couples’ HCT as well as from HIV counsellors indicated that the process of receiving couples’ HCT began with one of them taking the HIV test alone and then encouraging their partner to go with them on the subsequent visits to receive couples’ HCT (Table 2, issue# 3.1). This was particularly true in couples where at least one partner was HIV-positive. As indicated in the quotation below, people tend to go for HIV testing alone first *because they are not sure of their HIV status* and may not want to be shocked by unexpected results in front of their partners:

We found that in most cases, men first test for HIV alone and get to know that he is HIV negative and then goes to bring the wife/partner so that they test together. But they first test alone because they are not sure of their HIV status. He fears that if I first test with the wife and the results are not good; I rather not test together with her (KII with HIV Counsellor, Kasensero)

While some individuals did not report initial resistance from their partners when they requested them to test together with them, there was agreement that in the majority of cases, there was initial resistance from the approached partner (Table 2, issue# 3.2), but this waned with more discussion about the importance of couples’ HCT as well as through support from a professional counsellor:

My husband was the first to test for HIV. Okay he started testing before we were in the relationship. When we started a relationship, he requested me to go with him and test for HIV. But I refused … he insisted that we should go and test for HIV … So eventually, I accepted to go and test [with him]. We received the results together and the results showed that he was HIV positive and I was HIV negative (IDI with an HIV-negative female partner in an HIV-discordant relationship, Kasasa-Sanje)

One interesting case was that of a concordant HIV-positive couple in which the female partner tested alone first and went back to convince her male partner to test with her, after knowing her HIV-positive status. This woman indicated that while she was initially “threatened and scared” after learning her HIV-positive status, she soon gained the courage to convince her husband to go with her for couples’ HCT, after convincing the counsellor to keep quiet about her initial HIV-positive results. In a related case below, an HIV-negative partner in an HIV-discordant relationship narrates how she was able to overcome the initial frustration that came with finding out that she was in an HIV-discordant relationship (Table 2, issue#3.3). That she was able to overcome her emotions and gained courage to stay in a relationship that she now calls “good and stable” is another skill that women in similar situations can use to deal with the negative consequences associated with couples’ HCT:

[When he tested HIV positive and I was HIV negative] I felt so bad but the health workers counseled me and helped me to get strong. They [health workers] told me that I can stay in that relationship and I may not get the infection. They [health workers] advised us on how to have safe sex that will prevent me from getting HIV from my husband. So, we left and came back home … since then, our relationship is good and stable. We do our work together (IDI with an HIV negative female partner in an HIV-discordant relationship, Kasasa-Sanje)

The situation among concordant HIV-negative couples was reported to be calm and friendly, and HIV-negative couples did not report any negative consequences prior to or during the process of receiving couples’ HCT. Rather, these couples tended to describe their experiences as very enriching and fulfilling (Table 2, issue# 3.4), noting that “everything went on well” even after they received their HIV test results together:

We did not experience any problem when we sought HIV counseling and testing. Everything went on well starting from the counseling we received before they drew blood from us to screen it for HIV. Even when we received our HIV test results, we did not get any problem and everything went on well (IDI with an HIV-negative man in a concordant HIV-negative relationship, Buyamba)

#### Suggestions for improving couples’ HCT

When asked about how best we could improve couples’ HCT uptake in Rakai, participants identified six main suggestions including: 1) inviting couples to come for couple-specific meetings in which they can discuss the benefits associated with couples’ HCT (Table 2, issue# 4.1); 2) issuance of invitation letters or coupons to the couples, that is, to invite couples to come to a health facility to test for HIV together with their partners (Table 2, issue# 4.2); 3) promote couples’ HCT by going to people's homes (Table 2, issue# 4.3); 4) using religious leaders to reach out to their folks with messages on the importance of couples’ HCT (Table 2, issue# 4.4); 5) giving preferential treatment to couples that turn up for couples’ HCT as opposed to those who come as individuals (Table 2, issue# 4.5) and 6) using “expert” couples – couples that have been tested together and received their HIV test results together – to testify before other couples as to how they navigated the process leading to couples’ HCT, including how they dealt with the fears associated with couples’ HCT (Table 2, issue# 4.6). Couple-specific sessions were seen as a mechanism through which couples would not only be encouraged to test together but also learn about the benefits associated with couples’ HCT:

It would be good if men and women were given a health education program together so that the husband gets to know and the wife as well. If they are taught differently, they get to know things differently. But if they are put together they can know that we were taught this thing and so you work out that problem together as a couple. You should invite us both the man and woman and test us together for HIV and we receive the test results together as a couple. (FGD, women, never_received_HCT, Kasensero)

Across interviews, participants indicated that men tend to resist testing together with their partners. As a suggestion, participants called for specific sessions targeting men as key decision-makers to improve uptake of couples’ HCT. There was a belief that if men were convinced about couples’ HCT, it would be easier for both partners to test for HIV together since women are usually willing to test with their male partners, but male partners tend to dodge couples’ HCT, claiming that they “do not have time.” In trying to target men, participants advised that we should aim at holding shorter meetings that fit within the “limited” time that they have:

[…] if you are targeting men, you have to plan with them and agree on the suitable time to meet them. You have to make sure that the program takes the shortest time possible because in most cases, men don't have time. They don't have much time to sit and wait as they listen. But with the women, it is very easy. In general, the women are usually easy when you invite them for an education program, they sacrifice time and come and attend the education program (KII, counsellor, Kasensero)

Use of religious leaders in promoting couples’ HCT (Table 2, issue #4.4) was particularly cited because these leaders usually have a big following, and can thus incorporate messages on couples’ HCT during their summons. Since these leaders tend to command respect from their followers, such public promotions of couples’ HCT can help to improve uptake of couples’ HCT among populations reached with the messages. Participants suggested a need for sensitizing these leaders *about HIV so that they are in a better position to deliver that knowledge to their followers*.

## Discussion

This study of the motivations for and barriers to couples’ HCT uptake among married couples in Rakai district, southwestern Uganda, shows that couples’ HCT uptake is largely driven by individual- and relationship-based factors. These factors can be grouped into three categories, namely: 1) the need to know each other's HIV status, 2) the need to reduce mistrust between partners, and 3) the need to enrol into HIV care or identify a treatment companion in the event of an HIV-positive test result for one or both partners. These findings suggest that couples’ HCT can facilitate timely identification of HIV-positive couples who can be linked to HIV care immediately after their HIV sero-positivity is confirmed (7, 12) and this would not only improve their survival but also reduce their level of infectiousness.

Barriers to couples’ HCT uptake rotated around fears of the negative consequences associated with receiving couples’ HCT. These consequences included fear of violence or marital dissolution; fear of having one's infidelity confirmed before one's partner, especially in the event that one of the partners is HIV-positive while the other partner is not; and fear of receiving HIV-discordant or HIV-positive results. In turn, these fears caused some partners to claim that they did not have time to take the HIV test together with their partners, or – in the case of men – to ask their female partners to test for HIV in the hope that the female partners’ HIV test results would be the same as theirs, a phenomenon known as HIV testing by proxy (37).

Studies show that the uptake of couples’ HCT has, in some instances, been hampered by male partners who refuse to take an HIV test together with their spouses (38, 39). As documented in our study, female partners indicated that they were willing to test for HIV together with their male partners, but male partners tended to claim that they did not have the time to test with their spouses. There were also fears, especially among men, that testing together could inadvertently reveal hidden infidelity, especially if they tested HIV-positive when their partners were HIV-negative. Such revelations could have disastrous consequences on the couple and the relationship between partners. These findings are in agreement with findings from other studies (20, 23) (25) and provide further evidence to highlight the role of gender and power dynamics in influencing uptake of critical interventions by both men and women in long-term sexual relationships. For instance, Siu *et al*. (19) found that couples’ HCT seemed to threaten masculine esteem by exposing their extra-marital relations and consequently severing their relations with their female spouses. Despite these fears, there is evidence that targeting men to encourage them to test for HIV together with their partners can improve uptake of couples’ HCT (40), and a recent randomized trial has suggested that targeting men as a specific population sub-group could improve couples’ HCT uptake especially at antenatal clinics (41).

Among couples that had ever received couples’ HCT, and especially those where at least one of the partners was HIV-positive, we found that the process leading to the decision to receive couples’ HCT was not a straightforward one, with some partners opting to take a test alone before inviting their spouses to go along with them. These experiences were generally shared by individuals who lived in HIV-discordant and concordant HIV-positive couples, further emphasizing the fears that individuals initially had (prior to couples’ HCT) that prompted them to test alone first. What is pertinent is that despite the initial hesitation, there was agreement at the end of the process and both partners tested and received their HIV test results together. This suggests a need to empower individuals with skills necessary to successfully negotiate for couples’ HCT while emphasizing that the negotiation process is not straightforward and might require multiple attempts to succeed.

Participants identified several approaches to improve couples’ HCT uptake, some of which have been tried out in other settings. The use of invitation letters has been implemented in Rwanda, Zambia and South Africa, particularly to invite men to come to antenatal care clinics to test for HIV together with their pregnant partners (27, 42–44) but this has not been implemented among couples in the general population. Inviting couples for couple-specific sessions could be one way of reaching couples with messages pertaining to couples’ HCT, and although there is no documented literature on its efficacy, the use of couple-focused meetings could be one of the promising approaches to improve HIV testing uptake among couples. The role of men in influencing uptake of reproductive health services has been documented, particularly in family planning, PMTCT of HIV and other sexual and reproductive health programmes (45). This suggests that male-targeted interventions, including those that aim to improve men's awareness of the benefits of couples’ HCT, could help to improve uptake of couples’ HCT among married couples in Rakai district and elsewhere (13, 14) (46).

This study had a number of limitations. In the first place, we enrolled individuals who had previously been enrolled into an ongoing RCCS, and classified them as never tested, ever tested alone and ever tested together with a partner. There is a possibility that some of those classified as never tested, or tested alone could have tested as a couple from outside the RCCS testing facilities, and their opinions might have been influenced by this kind of testing. However, previous research in Rakai shows that up to 95% of individuals resident within the study communities cite the Rakai Health Sciences Program as the prime source of HCT services (35). The other limitation is that there have been a number of interventions in Rakai, including interventions to promote HIV testing among those that have never tested for HIV. These interventions have largely promoted individual rather than couples’ HCT but could still have had an effect on the knowledge of the benefits and motivations for couples’ HCT. However, since the purpose of the study was to capture motivations for, barriers to and suggestions for improving couples’ HCT uptake, the effect of prior knowledge of couples’ HCT on our study can only be assessed in a positive direction as it could point to critical areas that need to be followed up urgently, including the use of “expert” couples, to promote couples’ HCT uptake in Rakai or any other district of Uganda.

Despite these limitations, our study presents findings from a stratified population that yields people's motivations for and barriers to couples’ HCT as well as suggestions for improving couples’ HCT from the point of view of those who have never tested, those who have ever tested individually and those that have ever tested as a couple. We believe that this unique classification and the ability to obtain these factors across the different population sub-groups provides a more general overview of the issues inherent in couples’ HCT promotion in any other Ugandan population, despite the fact that Rakai district has had many studies and interventions that may make it less similar to other Ugandan settings.

## Conclusions

In conclusion, this study of the motivations for and barriers to couples’ HCT uptake in Rakai, Uganda, shows that couples’ HCT uptake is largely influenced by individual- and relationship-based factors. The need to know each other's HIV status, to reduce mistrust between partners and by implication, to link to HIV care or improve anti-retroviral therapy adherence if one or both partners were HIV-positive emerged as critical motivators for couples’ HCT uptake. Fear of the negative social consequences that may follow after partners have received HCT together was the single most important barrier to couples’ HCT uptake in this setting. Interventions aimed at increasing couples’ HIV testing should build on the motivations for couples’ HCT reported in this study while addressing the negative social consequences associated with couples’ HCT.

## References

[1] UNAIDS (2012). UNAIDS Report on the global AIDS epidemic.

[2] Chemaitelly H, Awad SF, Shelton JD, Abu-Raddad LJ. (2014). Sources of HIV incidence among stable couples in sub-Saharan Africa. J Int AIDS Soc.

[3] Wabwire-Mangen F, Odiit M, Kirungi W, Kisitu KD. (2009). Uganda: HIV modes of transmission and prevention response analysis. Final report, March 2009.

[4] Dunkle KL, Stephenson R, Karita E, Chomba E, Kayitenkore K, Vwalika C (2008). New heterosexually transmitted HIV infections in married or cohabiting couples in urban Zambia and Rwanda: an analysis of survey and clinical data. Lancet.

[5] Uganda Bureau of Statistics [UBOS], ICF International Inc (2012). Uganda Demographic and Health Survey.

[6] Desgrees-du-Lou A, Orne-Gliemann J. (2008). Couple-centred testing and counselling for HIV serodiscordant heterosexual couples in sub-Saharan Africa. Reprod Health Matters.

[7] Cohen MS, Chen YQ, McCauley M, Gamble T, Hosseinipour MC, Kumarasamy N (2011). Prevention of HIV-1 infection with early antiretroviral therapy. N Engl J Med.

[8] Allen S, Meinzen-Derr J, Kautzman M, Zulu I, Trask S, Fideli U (2003). Sexual behavior of HIV discordant couples after HIV counseling and testing. AIDS.

[9] Allen S, Serufilira A, Bogaerts J, Van de Perre P, Nsengumuremyi F, Lindan C (1992a). Confidential HIV testing and condom promotion in Africa. Impact on HIV and gonorrhea rates. JAMA.

[10] Allen S, Tice J, Van de Perre P, Serufilira A, Hudes E, Nsengumuremyi F (1992). Effect of serotesting with counselling on condom use and seroconversion among HIV discordant couples in Africa. BMJ.

[11] Were WA, Mermin JH, Wamai N, Awor AC, Bechange S, Moss S (2006). Undiagnosed HIV infection and couple HIV discordance among household members of HIV-infected people receiving antiretroviral therapy in Uganda. J Acquir Immune Defic Syndr.

[12] World Health Organization (WHO) (2012). Guidance on couples’ HIV testing and counseling including antiretroviral therapy for treatment and prevention in sero-discordant couples: recommendations for a public health approach.

[13] Farquhar C, Kiarie JN, Richardson BA, Kabura MN, John FN, Nduati RW (2004). Antenatal couple counseling increases uptake of interventions to prevent HIV-1 transmission. J Acquir Immune Defic Syndr.

[14] Becker S, Mlay R, Schwandt HM, Lyamuya E. (2010). Comparing couples’ and individual voluntary counseling and testing for HIV at antenatal clinics in Tanzania: a randomized trial. AIDS Behav.

[15] Tumwesigye E, Wana G, Kasasa S, Muganzi E, Nuwaha F. (2010). High uptake of home-based, district-wide. HIV counseling and testing in Uganda. AIDS Patient Care STDS.

[16] Koenig MA, Lutalo T, Zhao F, Nalugoda F, Wabwire-Mangen F, Kiwanuka N (2003). Domestic violence in rural Uganda: evidence from a community-based study. Bull World Health Organ.

[17] Porter L, Hao L, Bishai D, Serwadda D, Wawer MJ, Lutalo T (2004). HIV status and union dissolution in sub-Saharan Africa: the case of Rakai, Uganda. Demography.

[18] Njau B, Watt MH, Ostermann J, Manongi R, Sikkema KJ. (2012). Perceived acceptability of home-based couples voluntary HIV counseling and testing in Northern Tanzania. AIDS Care.

[19] Siu GE, Wight D, Seeley JA. (2014). Masculinity, social context and HIV testing: an ethnographic study of men in Busia district, rural eastern Uganda. BMC Public Health.

[20] DiCarlo AL, Mantell JE, Remien RH, Zerbe A, Morris D, Pitt B (2014). ‘Men usually say that HIV testing is for women’: gender dynamics and perceptions of HIV testing in Lesotho. Cult Health Sex.

[21] Lingappa JR, Lambdin B, Bukusi EA, Ngure K, Kavuma L, Inambao M (2008). Regional differences in prevalence of HIV-1 discordance in Africa and enrollment of HIV-1 discordant couples into an HIV-1 prevention trial. PLoS One.

[22] Larsson EC, Thorson A, Nsabagasani X, Namusoko S, Popenoe R, Ekstrom AM. (2010). Mistrust in marriage – reasons why men do not accept couple HIV testing during antenatal care- a qualitative study in eastern Uganda. BMC Public Health.

[23] Parker L, Pettifor A, Maman S, Sibeko J, Macphail C. (2014). Concerns about partner infidelity are a barrier to adoption of HIV-prevention strategies among young South African couples. Cult Health Sex.

[24] Mlay R, Lugina H, Becker S. (2008). Couple counselling and testing for HIV at antenatal clinics: views from men, women and counsellors. AIDS Care.

[25] Cummings B, Mengistu M, Negash W, Bekele A, Ghile T. (2006). Barriers to and facilitators for female participation in an HIV prevention project in rural Ethiopia: findings from a qualitative evaluation. Cult Health Sex.

[26] Byamugisha R, Tumwine JK, Semiyaga N, Tylleskar T. (2010). Determinants of male involvement in the prevention of mother-to-child transmission of HIV programme in Eastern Uganda: a cross-sectional survey. Reprod Health.

[27] Mohlala BK, Boily MC, Gregson S. (2011). The forgotten half of the equation: randomized controlled trial of a male invitation to attend couple voluntary counselling and testing. AIDS.

[28] Fylkesnes K, Sandoy IF, Jurgensen M, Chipimo PJ, Mwangala S, Michelo C. (2013). Strong effects of home-based voluntary HIV counselling and testing on acceptance and equity: a cluster randomised trial in Zambia. Soc Sci Med.

[29] Matovu JK, Denison J, Wanyenze RK, Ssekasanvu J, Makumbi F, Ovuga E (2013). Trends in HIV counseling and testing uptake among married individuals in Rakai, Uganda. BMC Public Health.

[30] Sweat M, Morin S, Celentano D, Mulawa M, Singh B, Mbwambo J (2011). Community-based intervention to increase HIV testing and case detection in people aged 16–32 years in Tanzania, Zimbabwe, and Thailand (NIMH Project Accept, HPTN 043): a randomised study. Lancet Infect Dis.

[31] Weinhardt LS, Carey MP, Johnson BT, Bickham NL. (1999). Effects of HIV counseling and testing on sexual risk behavior: a meta-analytic review of published research, 1985–1997. Am J Public Health.

[32] Janz NK, Becker MH. (1984). The health belief model: a decade later. Health Educ Q.

[33] Roura M, Watson-Jones D, Kahawita TM, Ferguson L, Ross DA. (2013). Provider-initiated testing and counselling programmes in sub-Saharan Africa: a systematic review of their operational implementation. AIDS.

[34] Wawer MJ, Gray RH, Sewankambo NK, Serwadda D, Paxton L, Berkley S (1998). A randomized, community trial of intensive sexually transmitted disease control for AIDS prevention, Rakai, Uganda. AIDS.

[35] Matovu JK, Kigozi G, Nalugoda F, Wabwire-Mangen F, Gray RH. (2002). The Rakai Project counselling programme experience. Trop Med Int Health.

[36] Mostyn B., Bressner JBM, Conta D (1985). The content analysis of qualitative research data: a dynamic approach. The research interview: uses and approaches.

[37] Morrill AC, Noland C. (2006). Interpersonal issues surrounding HIV counseling and testing, and the phenomenon of “testing by proxy”. J Health Commun.

[38] Ditekemena J, Koole O, Engmann C, Matendo R, Tshefu A, Ryder R (2012). Determinants of male involvement in maternal and child health services in sub-Saharan Africa: a review. Reprod Health.

[39] Morfaw F, Mbuagbaw L, Thabane L, Rodrigues C, Wunderlich AP, Nana P (2013). Male involvement in prevention programs of mother to child transmission of HIV: a systematic review to identify barriers and facilitators. Syst Rev.

[40] Hayes R, Ayles H, Beyers N, Sabapathy K, Floyd S, Shanaube K (2014). HPTN 071 (PopART): rationale and design of a cluster-randomised trial of the population impact of an HIV combination prevention intervention including universal testing and treatment – a study protocol for a cluster randomised trial. Trials.

[41] Orne-Gliemann J, Balestre E, Tchendjou P, Miric M, Darak S, Butsashvili M (2013). Increasing HIV testing among male partners. AIDS.

[42] Byamugisha R, Astrom AN, Ndeezi G, Karamagi CA, Tylleskar T, Tumwine JK. (2011). Male partner antenatal attendance and HIV testing in eastern Uganda: a randomized facility-based intervention trial. J Int AIDS Soc.

[43] Allen S, Karita E, Chomba E, Roth DL, Telfair J, Zulu I (2007). Promotion of couples’ voluntary counselling and testing for HIV through influential networks in two African capital cities. BMC Public Health.

[44] Wall K, Karita E, Nizam A, Bekan B, Sardar G, Casanova D (2012). Influence network effectiveness in promoting couples’ HIV voluntary counseling and testing in Kigali, Rwanda. AIDS.

[45] van Dyk AC. (2013). Client-initiated, provider-initiated, or self-testing for HIV: what do South Africans prefer?. J Assoc Nurses AIDS Care.

[46] Conkling M, Shutes EL, Karita E, Chomba E, Tichacek A, Sinkala M (2010). Couples’ voluntary counselling and testing and nevirapine use in antenatal clinics in two African capitals: a prospective cohort study. J Int AIDS Soc.

